# Research Priorities for Neglected Infectious Diseases in Latin America and the Caribbean Region

**DOI:** 10.1371/journal.pntd.0000780

**Published:** 2010-10-26

**Authors:** Jean-Claude Dujardin, Socrates Herrera, Virgilio do Rosario, Jorge Arevalo, Marleen Boelaert, Hernan J. Carrasco, Rodrigo Correa-Oliveira, Lineth Garcia, Eduardo Gotuzzo, Theresa W. Gyorkos, Alexis M. Kalergis, Gustavo Kouri, Vicente Larraga, Pascal Lutumba, Maria Angeles Macias Garcia, Pablo C. Manrique-Saide, Farrokh Modabber, Alberto Nieto, Gerd Pluschke, Carlos Robello, Antonieta Rojas de Arias, Martin Rumbo, Jose Ignacio Santos Preciado, Shyam Sundar, Jaime Torres, Faustino Torrico, Patrick Van der Stuyft, Kathleen Victoir, Ole F. Olesen

**Affiliations:** 1 Institute of Tropical Medicine, Antwerp, Belgium; 2 Universidad del Valle, Cali, Colombia; 3 Instituto de Higiene e Medicina Tropical/Universidade Nova de Lisboa, Lisbon, Portugal; 4 Instituto de Medicina Tropical ‘A. von Humboldt’, Lima, Peru; 5 Universidad Central de Venezuela, Caracas, Venezuela; 6 Fiocruz, Belo Horizonte, Brazil; 7 Universidad Mayor de San Simon, Cochabamba, Bolivia; 8 McGill University, Montreal, Canada; 9 Pontificia Universidad Católica de Chile, Santiago, Chile; 10 Instituto Pedro Kouri, La Havana, Cuba; 11 Centro de Investigaciones Biologicas (CSIC), Madrid, Spain; 12 National Institute for Biomedical Research, Kinshasa, the Democratic Republic of Congo; 13 EULARINET, Ministerio de Ciencia e Innovación de España, Madrid, Spain; 14 Universidad Autónoma de Yucatán, Yucatán, Mexico; 15 Drugs for Neglected Diseases initiative, Geneva, Switzerland; 16 Universidad de la República, Montevideo, Uruguay; 17 Swiss Tropical and Public Health Institute, Basel, Switzerland; 18 University of Basel, Basel, Switzerland; 19 Institut Pasteur de Montevideo and Facultad de Medicina, Montevideo, Uruguay; 20 Centro para el Desarrollo de la Investigacion Cientifica/Panamerican Health Organization, Asunción, Paraguay; 21 Universidad Nacional de La Plata, La Plata, Argentina; 22 Facultad de Medicina, Universidad Nacional Autónoma de México, México, D.F., México; 23 Banaras Hindu University, Varanasi, India; 24 Instituto de Medicina Tropical de la Universidad Central de Caracas, Caracas, Venezuela; 25 Institut Pasteur, Paris, France; 26 EU Commission, Brussels, Belgium; Swiss Tropical and Public Health Institute, Switzerland

## Background and Rationale

Global priorities for research in neglected infectious diseases (NIDs) can be assessed in different ways, but it is important to realize that regional priorities may significantly differ one from another. The region of Latin America and the Caribbean (LAC) is—along with Africa and Asia—more affected by NIDs than other regions of the world. Some of the Latin American NIDs are common to other continents, while others are very specific or disproportionately affect the Latin American region [1]–[3] (Table 1). Because of its huge ecological diversity, ongoing environmental changes, and massive migrations, LAC is also a catalyst for the (re-)emergence and spreading of NIDs, both inside and outside the subcontinent. Following a colloquium on NIDs in LAC held in Lima, Peru, between 12 and 14 November 2009, a thematic workshop was organized with the support of the European Commission (EC). It involved 29 scientists (16 from the Americas, two from the Democratic Republic of Congo and India, respectively, and nine from Europe) working on different NIDs and representing several research areas from basic to applied. This report summarizes the consensus comments of the expert group after oral and written consultation. It is envisaged that this document should stimulate a debate within the scientific community and serve as a recommendation for future actions by international or regional funding agencies in the area of NIDs in LAC.

**Table 1:** Examples of Some Major NIDs Affecting Latin America and the Caribbean.

	NIDs Affecting the Latin American, Caribbean, and Other Regions	NIDs with a Disproportionate Impact in the Latin American Caribbean Region
**PAHO-targeted NIDs**	Congenital syphilisLeprosyLymphatic filariasisNeonatal tetanusOnchocerciasisPlagueRabiesSchistosomiasisTrachoma	Chagas diseaseSoil-transmitted helminthiasis
**Other NIDs**	Buruli ulcerDengueVisceral leishmaniasis	BartonellosisEchinococcosisHantavirusHistoplasmosisLeptospirosisMuco-cutaneous leishmaniasis (Espundia)Paracoccidioidomycosis

## Priority NIDs and Major Research Gaps in Latin America and the Caribbean Region

**Figure 1:**
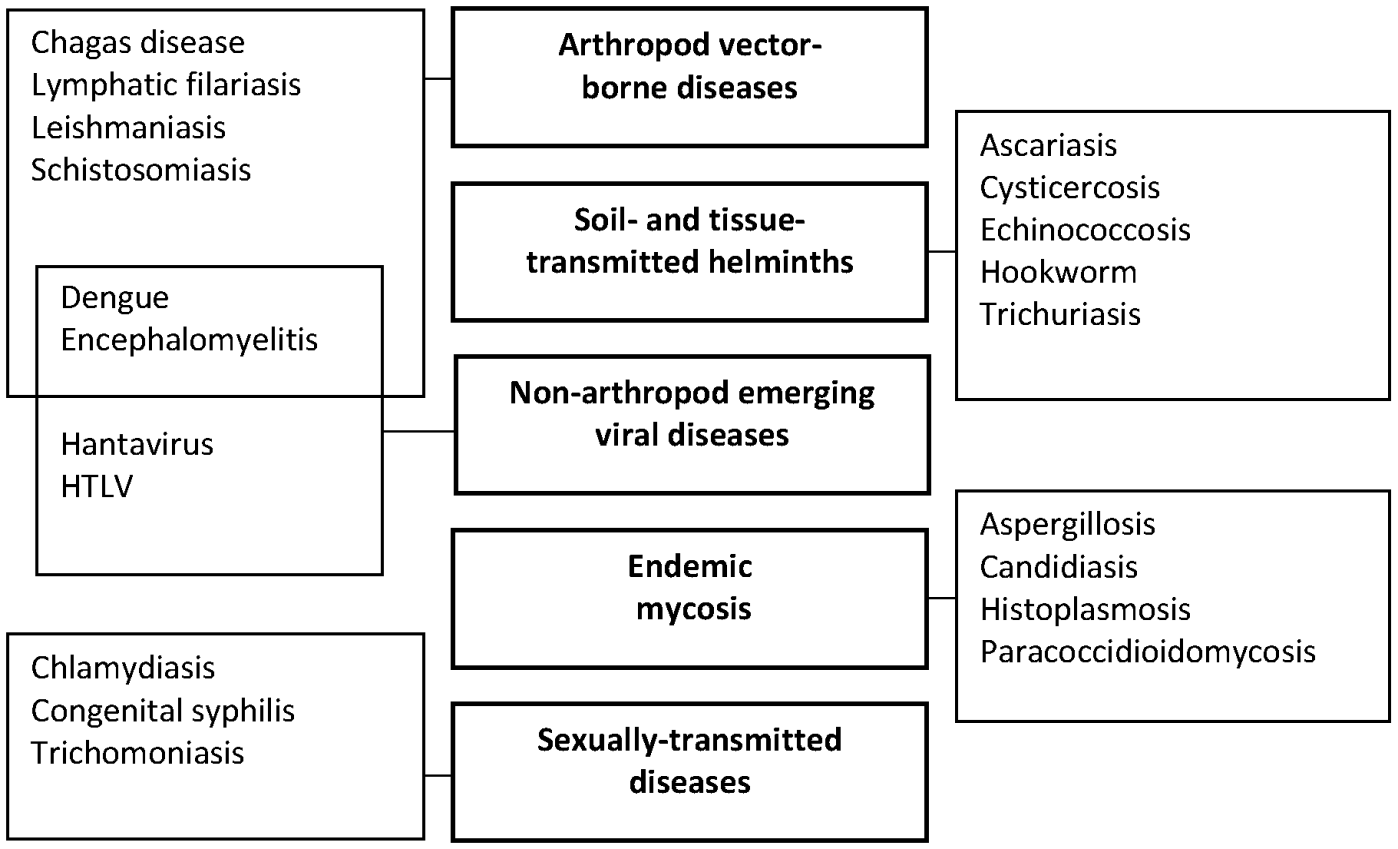
Five groups of diseases were identified as priorities for research in LAC, and examples of key diseases belonging to each group are indicated. Note: there is an overlap between the emerging viral diseases of regional importance and the arthropod vector-borne diseases.

Listing and priority setting for NID research is a matter of context, and should not be confused with the burden of the diseases themselves. The major NIDs in LAC in terms of disease burden (usually expressed in terms of disability-adjusted life years [DALYs] lost) have previously been listed on the basis of their prevalence and estimated DALYs [4], [5]. However, the prevalence of many NIDs could be significantly reduced with existing tools, making them a focus for improved public health service delivery rather than a research priority. The Pan American Health Organization (PAHO) has thus set a goal to eliminate or significantly reduce 12 NIDs in the Americas, before 2015, using existing tools [6]. In the present policy platform, the scientific panel focused on priority setting for regional NIDs from a research perspective. A rational way to prioritize the relevance and importance of research for the large and diverse group of NIDs could be to use specific indicators of poverty, mortality, and morbidity, but for several of these diseases bona fide data are lacking or unreliable. Furthermore, some groups in society, typically those in impoverished living conditions, are usually susceptible to more than one infection or disease, and therefore studies or control programs may need a wider scope [7]. The panel decided therefore to focus further discussions on groups of NIDs rather than on specific diseases (Figure 1). Similarly, the panel decided not to rank desired drugs, diagnostics, and vaccines, as there is an ongoing effort by (i) the World Health Organization (WHO) and the Special Program for Research and Training in Tropical Diseases (TDR) to identify global priorities for NIDs and (ii) the Pan American Health Organization (PAHO) to establish a local research agenda for the Latin American region.

Arthropod vector-borne diseases constitute a first and highly relevant group, especially because they are strongly poverty related and disease patterns are highly vulnerable to anthropogenic and environmental changes. In general, there is a lack of quality and affordable diagnostic tests available to existing facilities (central reference laboratories, peripheral laboratories, and point-of-care settings). Clinical evidence is often lacking for the treatments in use and innovation in drug development should be strongly supported. The role of asymptomatic infections in transmission should be elucidated, while tools for better epidemiologic surveillance and well-defined therapeutic strategies for the chronic carriers are needed. Epidemiologic studies, mainly on the competence and transmission capacity of (secondary) vectors, are needed, especially where environmental changes and disturbance of life cycles occur. Innovation in vector control tools and research on insecticide resistance is crucial in all vector control programs, while studies on social determinants are of special importance in relation to acceptance by local communities of delivery of control measures.

Soil- and tissue-transmitted helminths represent a second major group of poverty-related diseases; these infections are impediments to the physical and mental growth of children and a source of increasing social distress in LAC. Various studies in LAC have shown that soil-transmitted helminths (STHs) can be eliminated or reduced in cost-effective ways using existing tools [8]. However, the impact of control programs needs to be analyzed and studies on potential drug resistance need to be carried out in areas where only limited success was achieved. Identification and optimization of best practices should be supported, and linked to regional networking for research transfer and uptake into health policies and practice aimed at all levels of society, with an expected positive impact on health. Evaluation of the impact of chronic helminth infection on immunity against other concomitant infections and on vaccination efficacy is recognized as a critical research gap.

Third, endemic mycosis is caused by a number of pathogens, some of which are specific to the Latin American and Caribbean region. The well-established therapeutic and prophylactic regimens for bacterial infections enable longer survival of immunosupressed patients (ISPs) but have contributed to increased rates of fungal infections. Epidemiology of fungal infections in ISPs includes an increase in non-*albicans Candida* spp. and an increase in aspergillosis with its high mortality, which can reach up to 80% in some cases, despite treatments [9]. Therapeutic limitations associated with critically ill patients demand a deeper understanding of the interactions between fungal cells and the immune system of the host. Neutropenic, oncological ISPs under aggressive anti-tumoral chemotherapy and intensive care patients are highly susceptible to invasive fungal infections. A subset of such patients can develop infections more frequently, which may indicate that different host genotypes contribute to an increased resistance/susceptibility to fungal infections [10]–[12]. The main areas of research and development that need to be targeted are diagnosis and identification with new tools, epidemiologic determinants of infection, new therapeutic drugs, and interaction with other pathologies [13].

Fourth, emerging, non-arthropod-borne viral diseases of regional importance are poorly studied in terms of epidemiology and differential diagnosis. LAC as a region has a significant disease burden due to new and re-emerging viral diseases, especially zoonotic diseases, and their occurrence and burden of disease remain largely underestimated. For instance, the newly emerging HTLV-1 is rarely detected in Latin American and Caribbean countries, with the exception of Peru, although it has been shown to be the cause of significant morbidity in other areas of the world. A comparative study in LAC that includes several countries and regions would shed light on this issue.

The fifth and final priority identified was sexually transmitted diseases (STDs) other than HIV/AIDS. STDs constitute a complex of infections of different origins and pathogens that need regular updating on species and strain identification associated with early diagnosis, treatment, and drug resistance.

## Technological Strengths and Opportunities in Latin America and the Caribbean Region

A second approach taken for priority setting was to identify research and technological strengths and opportunities in LAC. Several research and technological networks already exist in the region, and these might constitute promising platforms for new cost-effective research actions (Table S1). This “top-down” approach also aimed to list domains in which capacity strengthening should be further undertaken.

Significant disparities exist in research and technological facilities among Latin American and Caribbean countries, with major research investments in countries like Brazil and Cuba, and major difficulties with brain drain in many countries, including those in Central America. Major disparities also exist within many countries, with some countries recognized internationally for the caliber of their research. Well-established regional institutions could therefore play a major role as focal points for South–South collaborations and capacity strengthening. The possibility of networking the major research institutes in LAC, much like Institut Pasteur has done in the past, should be entertained. PAHO has definitely offered opportunities for this in the past, as did WHO/TDR, and major donors such as the European Union should be encouraged to support such initiatives. At the same time, North–South partnerships should avoid the temptation to focus only on strong partner countries from the South, but should also consider weaker ones, where capacity strengthening would bring an added value. Among the specific research and technological strengths/opportunities identified by the panel in the region, the following deserve particular attention.

### Natural Products Research

The biodiversity found in LAC is one of the richest in the world, yet its potential as a source of new pharmaceuticals or agrochemicals has not been fully realized. The Latin America Network for Research on Bioactive Natural Compounds (LANBIO) was created to promote research on natural products in the region. These studies represent an important avenue to promote local research capacity and to generate new knowledge, as well as to attract resources for sustainable research activities. No single country has all the natural resources, manpower, and facilities required. Complementarity in regional natural products research activities is therefore one critical reason for the existence of LANBIO. However, there is a need to strengthen its capacity to allow for the research, production, and testing of candidate products to meet current international quality standards.

### Clinical/Epidemiologic Research

In spite of great limitations and disparities among countries, several excellent research centers have developed in the region. They have been pivotal in generating knowledge of endemic pathologies, providing highly specialized professional training, promoting multicentric and multinational basic as well as clinical and epidemiologic research, and incorporating new technologies and/or procedures. Therefore, numerous opportunities exist to expand and reinforce such achievements through strategic partnerships and associations with similar institutions in both developing and developed countries. Over the last few years, WHO and TDR have supported the training of scientists from disease-endemic countries, including Latin American and Caribbean countries, in both good laboratory practices (GLPs) and good clinical practices (GCPs). This has led to the establishment of a network of GLP trainers who can provide expert assistance in future research activities. WHO/TDR has also promoted the establishment of GLP-compliant laboratories that will enable the production of high-quality data for both non-clinical safety studies as well as for clinical and epidemiologic studies [14]. New research efforts can benefit from these advancements, which, in turn, will consolidate and strengthen further training initiatives in good practices related to social sciences, bioethics, project planning, and evaluation.

### Medical Entomology

LAC has prioritized the development of human resources on medical entomology (ME) in the past, but unfortunately this situation is changing. The biology and behavior of vector species, and even knowledge about which vectors are implicated in specific diseases, still remain undefined in many cases in LAC. Decisions about vector control are often based on extrapolations from other regions (Africa or Asia). Research on new and improved surveillance and control tools/strategies should be encouraged. LAC has acquired expertise in the control of specific NIDs, most notably Chagas disease. Elimination and control in several countries has been achieved through well-organized programs and regional discussions that have included a large number of stakeholders. Multidisciplinary research played a major role in paving the way for program launch, but also to monitor it over the time. The expertise accrued in terms of data exchange, standardization of technologies, training at different levels, usage of infrastructures, and transfer of sustainable technology can be applied to other diseases or to groups of diseases sharing similar aspects in their life cycle or clinical profile. Social and public health studies should be integrated, and here again the experience acquired with Chagas disease could easily be applied to other “social diseases” [15]. Even “successful” control strategies such as the Chagas disease control program could be further strengthened, as many challenges lie ahead for the control of vector transmission (for example, the presence of relevant sylvatic populations [16] or resistance to residual insecticides [17]). Similarly, non-chemical control, in general, needs further studying if we take into account the unsustainable and limited efficacy as well as the potential impact of pyrethroid-based pesticides on human health [18]. ME research in LAC needs capacity strengthening, in both managerial as well as technical issues, i.e., the use of geographic information system (GIS) and remote sensing technologies to study vector distribution [19], and molecular tools that help in the determination of vector species and vector incrimination, amongst others, without neglecting the basic skills of vector behavior studies. Integrated vector management (IVM) and translational research for vector control (i.e., how to translate research outputs into policy) should be encouraged, considering the similar challenges encountered among different arthropod-borne diseases. Collaboration between existing networks (Table S1) will encourage new partnered research and integrated strategies in vector control.

### Genomics and Bioinformatics

Early efforts in this research area have definitely helped countries in LAC to increase their scientific impact at the international level and to stop their brain drain [17], leading to a series of well-established networks (Table S1). The ongoing technological revolution in new high-throughput sequencing technologies represents a unique opportunity for LAC. On the one hand, this revolution will democratize a technology that, until now, has largely been reserved for large genome centers [20]. On the other hand, the new technologies offer the possibility to undertake parallel genome-wide sequencing of different strains representative of the clinical and phenotypic diversity of a given microorganism [21], [22]. This approach is likely to provide an unlimited source of information for epidemiology, diagnostics, tracking of drug resistance, drug and vaccine discovery, and pharmacogenomics, among others. Recognizing the huge biodiversity that exists in LAC, the strengthening of a new generation of genomic tools could provide considerable added value to the regional research environment.

### Biotechnology Incubators

A particular case of South–South partnership concerns small and medium enterprises (SMEs) in the biotechnology sector in LAC. The region has a small but growing number of countries with established biotechnology SMEs that would be in a position to contribute to the production of new drugs, diagnostic kits, and vaccines for NIDs. South–South collaboration would contribute to connect providers and users, including more developed and less developed research groups, countries, or disease foci. The inclusion of local SMEs in academic research collaborations could result in important cross-fertilization between the two sectors, and such public–private partnerships should be strongly encouraged by both private and public research funders. Although the latter is still new in most of LAC, there are leading institutions experienced in the transfer of technological development into products. Most of the products are geared to supply the needs of the Ministries of Health and research is still incipient in these facilities. Although a significant increase in biotech parks throughout LAC has occurred, there is still a large gap between the transfer of “prototypes” to production. Significant experience has been gained from Brazilian institutions such as FIOCRUZ and Instituto Butantan on product development and production. These institutes have been able to supply the country and export several of the basic vaccines and pharmaceuticals needed for the nation's health programs. However, there is still a need for an increase in interaction and transfer of technological development in health among the biotechs in Latin American and Caribbean countries and the available industrial parks in those countries. This process is crucial to position LAC at a significantly higher level of competitiveness in supplying the countries with pharmaceuticals and biologicals in all health fields, including NIDs.

## Cross-Cutting Issues and Collaborative Research

Research activities can be driven by different factors, and supported in varying degrees by governments, funding agencies, economic interests, patients' organizations, and research communities themselves. The desire to overcome a major public health problem may lead to needs-driven research. Opportunity-driven research can come from an interest in potential economic gain, or the examination of readily available data from routinely collected administrative databases or from a particular technological or scientific situation. Hypothesis-driven research is normally initiated by researchers themselves from scientific curiosity or in a search for basic biological mechanisms. The present panel of experts suggested that the ideal research consortium on NIDs in LAC should be a blend of needs-, hypothesis-, and opportunity-driven research. This strongly depends on a number of factors ranging from infrastructures to sustainability and trained local personnel so that scientific autonomy and excellence become the rule in LAC, as many countries have already shown [13].

The need for better interaction between different scientific areas, which range from social aspects to vector biology and control, immunogenetics, genomics, biotechnology, community participation, and dissemination of information, was strongly recommended. This has also previously been highlighted in the frame of the LAC–European Union Cooperation (ALCUE) Health workshop [23]. Major developments in biotechnology have not necessarily been applied to improve health conditions, due to many different reasons, including a lack of communication between the research environment and policy-makers or the lack of a proper needs-based agenda; at the same time, major social changes associated with overpopulated urbanized areas, major migrations, and other upheavals have considerably diminished the proper use or application of control programs. When possible and relevant, research efforts should be coordinated to enhance targeted, cost-effective objectives. In this context, strengthening research on prediction, identification, modeling, and surveillance of NIDs is an example of a cross-cutting priority. The aim would be to develop a network of scientists and a battery of tools allowing (i) a better surveillance of the (re-)emergence and spreading of LAC NIDs and their associated risks, and (ii) integrated disease management. An interdisciplinary approach should be followed. Research in public health, health systems, and epidemics preparedness should constitute one of the core elements, among others, of the identification of better parameters to quantify the burden of disease. A clinical component would also be needed and should aim, among others, at standardized case definitions: from asymptomatic to the different clinical manifestations of the respective diseases. The laboratory component should standardize the existing tools for the detection and typing of pathogens and apply them in a few model situations; specific attention should be paid to the performances of the assays in asymptomatic individuals. Genomic and post-genomic approaches might be considered for a high-throughput characterization of pathogen diversity. Another laboratory component should focus on the vectors (if any) with a special emphasis on their ecology; the search for potential secondary vectors and their capacity for transmission should be encouraged. Environmental sciences and mathematical modeling should interact closely with the previous domains in order to provide an integrated package of validated surveillance tools that would be useful to health authorities. The research consortia should involve members of different existing regional networks and pay particular attention to promote dissemination of information (at all levels).

## Conclusion

Research priorities for NIDs are strongly influenced by social and regional contexts. While global priorities for NID research are recognized, it is important to realize that regional priorities may be significantly different. We have identified the following key areas for research in NIDs for LAC: arthropod vector-borne diseases, soil- and tissue-transmitted helminths, endemic mycosis, emerging non-arthropod-borne viral diseases, and STDs other than HIV/AIDS. Research should build on existing strengths (such as networks that are already functional), but also promote further North–South as well as South–South partnerships. Particular opportunities are identified for research in natural products, clinical and epidemiologic research, ME, genomics/bioinformatics, and applied biotechnology. Clinical research should be linked with public health as well as basic research. ME should be encouraged through a combination of field studies and basic research, including molecular studies in the vectors and in the response to insecticides. Genomics with special emphasis in bioinformatics and deep sequencing could provide innovative information on the diversity of pathogens present in the region. The involvement of Southern SMEs would be an opportunity to provide local solutions to neglected problems.

Policy-makers, private charities/foundations, and national and international funding agencies are increasingly supporting research in NIDs. National Councils for Science and Technology exist in practically all Latin American and Caribbean, countries and most provide some funding opportunities for NID research, but at very different levels of magnitude. Many research teams in LAC are therefore dependent on collaborations with international research teams and funders. This can play an important role in priority setting by allocating attention, funding, and other resources to some activities, but not to others. Continued dialogue between research funders and policy-makers on the one hand, and the LAC research community on the other, is therefore pivotal in establishing an intelligent research agenda.

## Supporting Information

Table S1 Examples of networks and initiatives for regional scientific collaboration in LAC with relevance to NIDs
(0.05 MB DOC)
