# Supplementary material for: Research Priorities for Neglected Infectious Diseases in Latin America and the Caribbean Region
Source: PLoS Negl Trop Dis. 2010 Oct 26;4(10):e780. doi: 10.1371/journal.pntd.0000780 (PMC2964298; doi:10.1371/journal.pntd.0000780)
Supplement: Table S1 — Examples of networks and initiatives for regional scientific collaboration in LAC with relevance to NIDs (0.05 MB DOC) [file pntd.0000780.s001.doc]

**Table S1**: Examples of networks and initiatives for regional scientific collaboration in LAC with relevance to NIDs

| **Acronym** | **Name** | **Countries involved** | **Activity area** | **Website** |
| --- | --- | --- | --- | --- |
| AMSUD | AMSUD-Pasteur | Argentina, Brazil, Chile, Paraguay, Uruguay | Supports research cooperation between LAC and Europe, in particular the Pasteur Institute in Paris | http://www.pasteur.fr/pasteur/international/amsud/amsud.html |
| BIOTECSUR | BIOTECSUR | Argentina, Brazil, Paraguay, Uruguay | Establish and subsidize biotechnology projects between the four Mercosur countries | http://www.biotecsur.org/ |
| CABBIO | Center for Argentinian-Brazilian Biotechnology (Centro Argentino Brasileño  de Biotecnología) | Argentina, Brazil | Supports collaborations between research groups in Brazil and Argentina | http://www.mincyt.gov.ar/cabbio2.htm |
| CYTED | Latin American Science & Technology Development Programme (Programa Iberoamericano De Ciencia Y Tecnologia Para El Desarrollo) | Argentina, Bolivia, Brazil, Chile, Colombia, Costa Rica, Cuba, Ecuador, El Salvador, Guatemala, Honduras, Mexico, Nicaragua, Panama, Paraguay, Peru, Dominican Republic, Uruguay, Venezuela (and Spain, Portugal) | Supports integration of scientific communities in Latin America, and enables their cooperation with the European Union | http://www.cyted.org/ |
| LANBIO | LA Network for Research on Bioactive Natural Compounds (Red Latinoamericana para la Investigación de Compuestos Naturales Bioactivos) | Latin American countries | Promote natural product research in Latin America | http://abulafia.ciencias.uchile.cl/lanbio/ |
| LAND | Latin American Network for Dengue Control (Red Latinoamericana para el control de la dengue) | Brazil, Cuba, Guatemala, Mexico, Venezuela | Unify and increase the information exchange and advice on the design, field study, data analysis and policy conclusions of TDR and regional dengue control research programs | http://apps.who.int/tdr/svc/publications/tdrnews/issue-80/innovative-vector-control-research |
| RAVREDA | The Amazon Network for the Surveillance of Antimalarial Drug Resistance (Red Amazónica de Vigilancia de la Resistencia a los Antimaláricos) | Bolivia, Brazil, Colombia, Ecuador, Guyana, Peru, Suriname, Venezuela | Medical entomology | http://www.paho.org/English/ad/dpc/cd/ravreda-ami.htm |
| RELAB | Latin American Network of Biological Sciences (Red Latinoamericana de Ciencias Biológicas) | Argentina, Bolivia, Brazil, Chile, Colombia, Costa Rica, Cuba, Ecuador, Honduras, Mexico, Panama, Paraguay, Peru, Uruguay, Venezuela | Supports regional scientific integration through grants to research projects | www.relab.ws/SobreRelab.php |
| RELCOV | Latin American Vector Control Network (Red Latinoamericana de Control de Vectores) | Latin American institutions with interest in vector control | Medical entomology with emphasis on vector control | http://www.relcov.org/ |
| RIB | Iberoamerican Network for Bioinformatics (Red Latinoamericana de Bioinformatica) | Argentina, Bolivia, Brazil, Chile, Colombia, Cuba, Mexico, Paraguay, Uruguay, Venezuela (and Spain). | Education, research and service on bioinformatics, and collaboration with other networks | <http://rib.cecalc.ula.ve/> |
| SMSP | Mesoamerican Health System Initiative (Sistema Mesoamericano de Salud Pública) | Belize, Colombia, Costa Rica, El Salvador, Guatemala, Honduras, Mexico, Nicaragua, Panama | Support to technical cooperation in public health | http://portal2.sre.gob.mx/mesoamerica/ |
| UNU-BIOLAC | United Nations University Programme for Biotechnology for Latin America and the Caribbean (Programa de la Universidad de las Naciones Unidas/Biotecnología para América Latina y El Caribe) | Latin America and Caribbean countries | The program supports networks on bio-ethics, bio-safety and bioinformatics | http://www.unu-biolac.com/ |
